# Response to Plasmapheresis Measured by Angiogenic Factors in a Woman with Antiphospholipid Syndrome in Pregnancy

**DOI:** 10.1155/2015/123408

**Published:** 2015-08-27

**Authors:** Karoline Mayer-Pickel, Sabine Horn, Uwe Lang, Mila Cervar-Zivkovic

**Affiliations:** ^1^Department of Obstetrics and Gynecology, Medical University of Graz, Graz, Austria; ^2^Department of Nephrology, Medical University of Graz, Graz, Austria

## Abstract

An imbalance of angiogenic and antiangiogenic placental factors such as endoglin and soluble fms-like tyrosine kinase 1 has been implicated in the pathophysiology of preeclampsia. Extraction of these substances by plasmapheresis might be a therapeutical approach in cases of severe early-onset preeclampsia. *Case Report*. A 21-year-old primigravida with antiphospholipid syndrome developed early-onset preeclampsia at 18 weeks' gestation. She was treated successfully with plasmapheresis in order to prolong pregnancy. Endoglin and sflt-1-levels were measured by ELISA before and after treatment. Endoglin levels decreased significantly after treatment (*p* < 0.05) and showed a significant decrease throughout pregnancy. A rerise of endoglin and sflt-1 preceded placental abruption 4 weeks before onset of incident. *Conclusion*. Due to the limited long-term therapeutical possibilities for pregnancies complicated by PE, plasmapheresis seems to be a therapeutical option. This consideration refers especially to pregnancies with early-onset preeclampsia, in which, after first conventional treatment of PE, prolongation of pregnancy should be above all.

## 1. Introduction

The antiphospholipid syndrome (APS) is an autoimmune disease characterized by the presence of antiphospholipid antibodies (anticardiolipin antibodies (ACLA), lupus anticoagulants (LA), and ß2-glycoprotein) in the maternal circulation. These antibodies are associated with arterial and venous thromboses and with adverse obstetric outcomes such as recurrent fetal loss, intrauterine growth restriction (IUGR), intrauterine fetal death (IUFD), and preeclampsia [1].

Preeclampsia complicates 1% to 7% of all pregnancies and is a leading cause of pregnancy-associated mortality and morbidity in developed countries. Mechanisms suggested to explain APS include thrombosis, vascular and endothelial inflammation, and an imbalance of angiogenic and antiangiogenic placental factors such as endoglin and soluble fms-like tyrosine kinase 1 (sflt-1) [2, 3]. In APS, anti-endothelial cells antibodies lead to endothelial cell injury and apoptosis. The underlying pathophysiology of this disease suggests an imbalance of an angiogenic substances associated with endothelial dysfunction [4, 5].

Treatment options for APS, especially in early gestation, are limited and based on low-dose aspirin and low-molecular-weight heparin. Severe cases have been treated with intravenous immunoglobulins (IVIG), corticosteroids, antimalarials, TNF-targeted therapies, and immunomodulatory agents such as pentoxifylline [6].

There is a rationale for extracting of antiangiogenic markers, particularly aPl, via plasmapheresis in patients with severe early-onset preeclampsia [7]. Plasmapheresis has been used successfully in pregnancy for preeclampsia and HELLP-syndrome [8–12], as well as APS [13–19], and has been reported being safe during pregnancy [20].

We report a case of a woman with APS who developed severe preeclampsia at 19-week gestation and was successfully treated with plasmapheresis.

## 2. Case Report

A 21-year-old primigravida with a 4-year history of APS and a history of deep vein thrombosis was admitted to our department at 18 + 3 weeks' gestation with preeclampsia. Thrombophilia screening showed high titers of aPl: Lupus-aPTT: 2 sec (normal: −41 sec); Lupus-LA1: 78.2 sec (normal: −45 sec); Lupus ratio: 2, 24 (normal: −1.30). Titers of ACLA and ß2-glycoprotein were normal: ACLA-screening: 4, 9 U/mL (normal 0, 0–10, and 0 U/mL); ß2-glycoprotein-screening: 7.9 U/mL (normal: < 10,0 U/mL). Antiphospholipase-a2-receptor antibodies were negative.

The patient was treated with low-molecular-weight heparin (LMWH) (2 × 60 mg enoxaparin) from beginning of pregnancy and received aspirin (100 mg/d).

At 19 weeks, the patient was admitted to the hospital because of suspected preeclampsia. Fetal growth and Doppler studies were normal, with a bilateral notch of the uterine artery. The patient reported pain in the right and left upper abdomen; she denied headache and blurred vision. Platelets were normal (154.000 G/L, normal: 140–440.000 G/L) as was uric acid (2.5 mg/dL, normal: 2.4–5.7 mg/dL). LDH (280 U/L, normal: 120–240 U/L); GPT (110 U/L, normal: −30 U/L); and GOT (94 U/L, normal: −35 U/L) were increased. There was mild proteinuria (330 mg/24 hours). Preeclampsia as confirmed and magnesium sulfate were started. Because of the early onset of the disease, thenormal fetal biometry, and the lack of fetal distress, we decided to recommend plasmapheresis with the intention of prolonging pregnancy.

The first plasmapheresis was performed at 19 weeks of gestation. Clinical symptoms improved immediately; GOT and GPT (GOT: 39 U/L; GPT: 48 U/L) and aPl (Lupus apTT: 20, Lupus-LA1: 45.2; Lupus ratio: 1.50) decreased. Subsequently plasmapheresis was performed at weekly intervals.

Plasmapheresis was carried out via a catheter placed in the right jugular vein. A plasma filtration technique with Hemaplex BT 900/A Filters (Dideco, Mirandola, Italy) was used. During each treatment session, 3 liters of plasma was removed and continuously substituted with 3,0 liters of solvent-detergent treated standardized pooled human plasma (Octaplas, Octapharma Vienna, Austria). Anticoagulation therapy during plasmapheresis consisted of 3000 IU (international units) heparin administered as intravenous bolus and 1500 IU heparin per hour as continuous infusion.

Shortly before and after every course blood samples were collected and centrifuged by 800 rpm for 10 minutes, sera were portioned in 200 *μ*L aliquots and stored at −80°C until measurement. Heparin is known to release sflt-1 levels into the maternal circulation in vitro and in vivo; therefore, blood samples were also collected one day after plasmapheresis.

A commercial ELISA kit (R&D Systems Inc., Minneapolis, USA) was used for assaying endoglin according to the manufacturer's protocol.

A commercial ELISA kit (Roche Diagnostics GmbH, Mannheim, Germany) was used for measuring sflt-1 and plgf according to the manufacturer's protocol.

**Figure 1:**
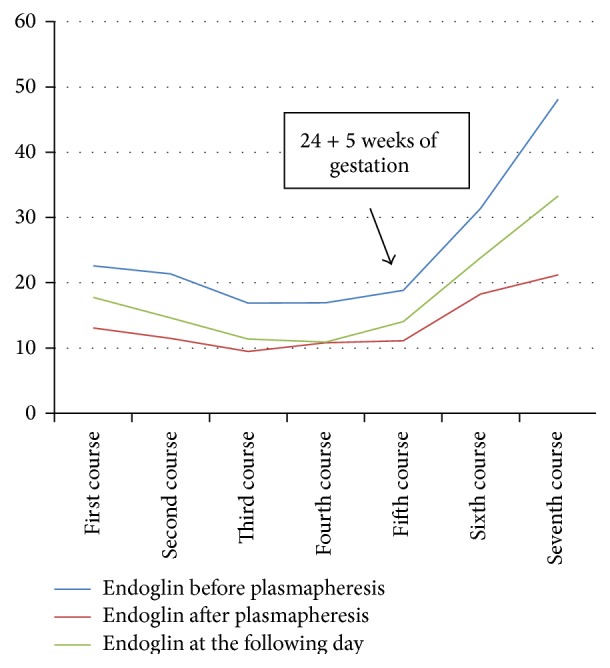
Endoglin, sflt-1, and plgf levels measured throughout pregnancy.

**Figure 2:**
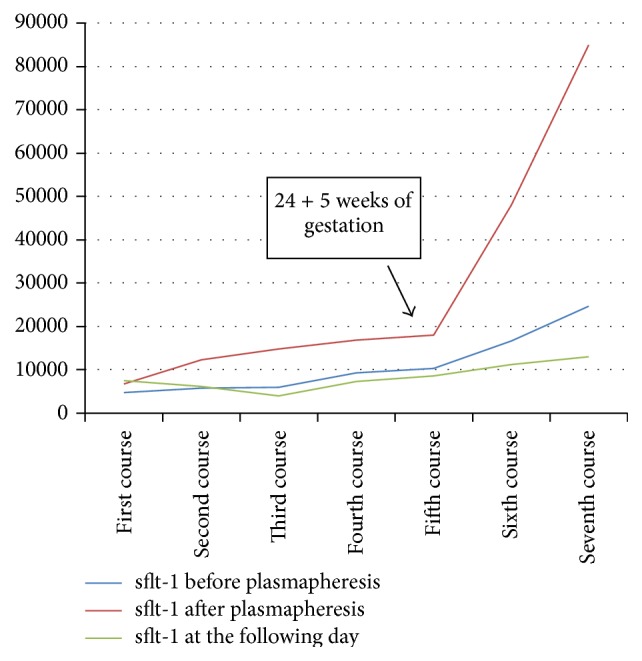
sflt-1, measured immediately before and after treatment and at the following day.

**Figure 3:**
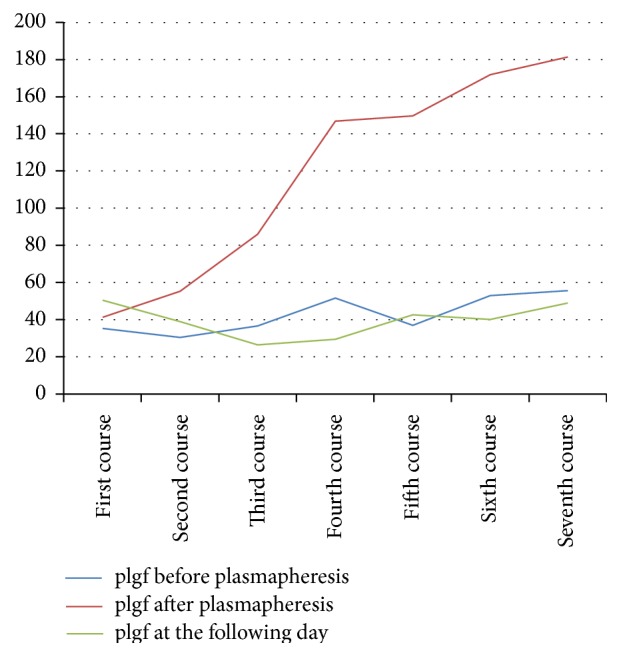
plgf, measured immediately before and after treatment and at the following day.

Mean endoglin levels decreased significantly from 25.15 ± 11.3 ng/mL (normal: 2.54–7.06 ng/mL) before to 13.6 ± 4.4 ng/mL after plasmapheresis (*p* < 0.05) (Table 1). sflt-1 levels (11042 ± 7213 pg/mL) increased after plasmapheresis (4742 ± 28112 pg/mL) due to the effect of heparin releasing sflt-1 into the maternal circulation (Table 1). However, sflt decreased the following day (8230 ± 3044 pg/mL, *p* = 0.36) (Table 1). Mean plgf levels (42 ± 10 pg/mL) also increased after plasmapheresis (118 ± 57 pg/mL) and decreased the following day (39 ± 9 pg/mL, *p* = 0.5) (Table 1). Endoglin measurements showed a decreasing trend over time, with a nadir (16.88 ng/mL before plasmapheresis and 9.45 ng/mL after plasmapheresis) at 20 + 5 weeks of gestation (Figure 1). sflt-1 levels measured before plasmapheresis as well one day after plasmapheresis showed a decreasing trend until the fifth course (24 + 5 weeks of gestation) (Figure 2). plgf immediately after plasmapheresis showed an increasing trend throughout gestation. The measurements before plasmapheresis as well as at the following day showed a similar trend (Figure 3). All courses were well tolerated; the patient was asymptomatic. Blood pressure was normal, as were platelets, LDH, uric acid, GPT, and GOT.

At 24 + 5 weeks, endoglin and sflt-1 increased (Table 1). The patient was asymptomatic. Laboratory workup showed mild thrombocytopenia (133.000 G/L). Blood pressure was normal. Sonography showed normal fetal growth; Doppler studies of umbilical, cerebral artery, and ductus venosus were normal. There were no signs of placental abruption. Three days after the seventh plasmapheresis, severe vaginal bleeding was noted and an emergency caesarean section was performed. During the procedure, full abruption of the placenta could be noted. There were no signs of coagulopathy; vital signs were stable. A female preterm in stable conditions was delivered (830 g, APGAR: 8/9/9; pH: 7.37).

The patient was transferred to the intensive care unit in stable conditions for observation and retransferred after 2 days. The postoperative/postpartum period was without any complications; endoglin and sflt-1 and plgf and sflt-1/plgf ratio levels returned to normal values (Table 1). The patient could be dismissed after 2.5 weeks. The infant was discharged home at 2 months in stable condition.

## 3. Discussion

We describe a pregnant woman with APS who developed early-onset preeclampsia at 18 + 3 weeks' gestation and who was treated with plasmapheresis and developed placental abruption at 27 + 5 weeks. Endoglin levels as well as sflt-1 at time of admission were increased.

Measurements of endoglin showed a significant decrease after plasmapheresis as well as a decreasing trend throughout gestation until the fifth course (24 + 5 weeks of gestation) and 3 weeks before placental abruption. These findings confirm the involvement of endoglin in the pathophysiology of PE [21–31], as well as the obvious role of endoglin as marker for placental abruption [32].

Heparin likely has no effect on circulating factors of endoglin [33]. This finding is in contrast to sflt-1, which is known to be released into the maternal circulation by heparin [34, 35]. However, a recent study of the effect of heparin on circulating levels of sflt-1, sEng, and plgf in pregnant women who required anticoagulation therapy showed no differences of the levels of sflt-1 and sEng between women who received heparin and the control group. Also treatment with heparin was associated with increased maternal circulatory levels of plgf and a decreased sflt-1/plgf ratio [36].

Endoglin is a transmembrane glycoprotein that acts as a coreceptor for transforming growth factor-*β*. Endoglin is highly expressed on cellular membranes of the vascular endothelium and on the syncytiotrophoblast [37–40]. It is involved in angiogenesis and has a major role in maintaining vascular tone [41, 42]. Its soluble circulating form (sEng), produced through the proteocleavage of the placental membrane-bound form, is an antiangiogenic factor implicated in the pathogenesis of PE and HELLP syndrome [22]. Placental tissue expression of sEng is upregulated in patients with PE.

Plasmapheresis is not a standard treatment for APS-related pregnancy complications [43] but it has been used successfully in pregnancies with APS [13–19]. The rationale is the removal of aPl and proinflammatory and -coagulatory markers, adhesion molecules, vasopressive factors, and atherogenic lipoproteins. The goal is to improve maternal endothelial function, prevent thrombosis, and increase placental perfusion with consecutive impaired trophoblast invasiveness and placentation. This hypothesis has been recently confirmed in a pilot study of nine preterm preeclamptic women [9]. In our patient, the immediate response and the evident removal of endoglin support the use of plasmapheresis for treatment of pregnancies with PE, especially in early gestation, as well as pregnancies with APS to improve obstetric outcome.

Bontadi et al. reported a significant therapeutic decrease of aPl-antibodies (ACLA and ß2-glycoprotein) in 3 women successfully treated with plasma exchange and immunoadsorption as a second-line therapy in APS [15]. A retrospective European multicentre study found that certain pregnant women with APS (thrombosis and triple antiphospholipid autoantibody positivity) who received additional treatment including apheresis had a significantly higher live birth rate than controls receiving conventional therapy alone [19].

Thadhani et al. demonstrated the concept of removing pathogenic circulating factors with plasma exchange in 2011. The authors treated five women with very preterm PE with dextran sulfate cellulose apheresis. This reduced circulating sflt-1 levels and proteinuria in a dose-dependent manner and stabilized blood pressure without apparent adverse events. sflt-1 plasma levels decreased by 20%–30% [8]. Blaha et al. showed a significant decrease of soluble endoglin after extracorporeal elimination in 11 patients with severe familial disorders of lipid metabolism. They suggested that endoglin might serve as a marker for evaluation of treatment efficacy [44].

Plasmapheresis may be a therapeutic option particularly for pregnancies with early-onset preeclampsia. Only a few authors have described plasmapheresis as treatment for PE, mostly in case reports with diverse success [45–47].

The patient described in the present report showed a decrease of mean sflt-1 levels 1 day after plasmapheresis and a decrease of mean sflt-1 levels until 24 + 5 weeks, suggesting that treatment with LMWH itself might not influence the circulatory levels of sflt-1 (Yinon et al.). Additionally the authors noted a rerise of sflt-1, especially after the fifth course (24 + 5 weeks of gestation). These changes of sflt-1 levels might be associated with the development of placental abruption [32].

To our knowledge, this is the first report of a significant decrease of endoglin after plasmapheresis in a pregnant woman with APS and early-onset preeclampsia and placental abruption. However, several therapeutical approaches such as prophylactic apheresis or IVIG beginning early in the pregnancy, rather than starting at the onset of complications, could be an optional treatment, also to prevent complications of obstetric APS, including preeclampsia.

## Figures and Tables

**Table 1:** 

Gestational age (weeks)	Plasmapheresis	Endoglin	sflt-1	plgf	Ratio
18 + 5	Before	22,57	4755	35,15	135,02
	After	13,09	6772	41,19	164,34
	Following day	17,73	7512	50,46	135,25

19 + 5	Before	21,33	5714	30,35	188,27
	After	11,46	12264	55,31	322,21
	Following day	14,62	6109	38,87	160,29

20 + 5	Before	16,88	5983	36,47	164,05
	After	9,45	14801	85,94	174,21
	Following day	11,38	3989	26,35	153,55

22 + 5	Before	16,92	9325	51,57	180,82
	After	10,78	16850	146,9	115,36
	Following day	10,89	7241	29,4	249,54

24 + 5	Before	18,81	10304	36,87	279,46
	After	11,12	18040	149,7	121,73
	Following day	14,05	8563	42,54	203,01

26 + 5	Before	31,38	16600	52,87	313,97
	After	18,27	48045	171,9	280,29
	Following day	23,81	11209	40	265,57

27 + 5	Before	48,15	24618	55,62	442,61
	After	21,18	>85000	181,3	468,25
	Following day	33,28	12988	48,96	270,52

*14.12.2013*	*Caesarean section due to placental abruption*				

16.12.2013	2 days postpartum	10,15	1190	16,76	71

23.12.2013	One week postpartum	4,65	188	18,57	10,12
